# Validation of cardiovascular diagnoses in the Greenlandic Hospital Discharge Register for epidemiological use

**DOI:** 10.1080/22423982.2017.1422668

**Published:** 2018-01-31

**Authors:** Maria Tvermosegaard, Pernille Falberg Rønn, Michael Lynge Pedersen, Peter Bjerregaard, Inger Dahl Pedersen, Christina Viskum Lytken Larsen, Marit Eika Jørgensen

**Affiliations:** ^a^ Centre for Health Research in Greenland, National Institute of Public Health, Faculty of Health Science, University of Southern Denmark, Copenhagen, Denmark; ^b^ Steno Diabetes Centre Copenhagen, Gentofte, Denmark; ^c^ Arctic Research Centre, Aarhus University, Aarhus, Denmark; ^d^ Greenland Centre of Health Research, University of Greenland, Nuuk, Greenland; ^e^ Queen Ingrid Primary Health Care Centre, Nuuk, Greenland

**Keywords:** Cardiovascular disease, diagnoses, Inuit, Greenland, register

## Abstract

Cardiovascular disease (CVD) is one of the leading causes of death worldwide. In Greenland, valid estimates of prevalence and incidence of CVD do not exist and can only be calculated if diagnoses of CVD in the Greenlandic Hospital Discharge Register (GHDR) are correct. Diagnoses of CVD in GHDR have not previously been validated specifically. The objective of the study was to validate diagnoses of CVD in GHDR. The study was conducted as a validation study with primary investigator comparing information in GHDR with information in medical records. Diagnoses in GHDR were considered correct and thus valid if they matched the diagnoses or the medical information in the medical records. A total of 432 online accessible medical records with a cardiovascular diagnosis according to GHDR from Queen Ingrid’s Hospital from 2001 to 2013 (n=291) and from local health care centres from 2007 to 2013 (n=141) were reviewed. Ninety-nine and ninety-two percent of discharge diagnosis in GHDR from Queen Ingrid’s Hospital and local health care centres were correct in comparison with diagnoses in the medical record indicating valid registration practice. The correctness of cardiovascular diagnoses in GHDR was considered high in terms of acceptable agreement between medical records and diagnoses in GHDR. Cardiovascular diagnoses are valid for epidemiological use.

## Background and introduction

Cardiovascular disease (CVD) includes diseases of the heart and blood vessels, such as ischemic heart disease, stroke and myocardial infarctions. CVD is the leading cause of death worldwide according to the World Health Organization, with ischemic heart disease and stroke as the top 2 causes of death, respectively [1]. Public health recommendations for CVD among Inuit in Greenland are a debated subject because an understanding of a low cardiovascular burden among Inuit adhering to traditional marine foods has reigned for more than 70 years [2]. This understanding is supported by studies finding that the Inuit diet rich on n-3 fatty acids protects against CVD [3,4]. Nonetheless, the notion of a lower cardiovascular susceptibility is not based on systematic studies. Cross-sectional studies from the last half century have shown inconsistent findings with both lower [5], similar [6–8] and higher prevalence [9,10] of CVD among Inuit in Greenland and Canada compared to western countries. However, the above mentioned studies are based on intermediate risk factors such as lipid levels, blood pressure, electrocardiography findings, questionnaires or estimates of mortality. These may reflect differences in treatment standards, use of International Classification of Disease codes, diagnostic practice or differences in misclassification rather than actual CVD incidence.

Valid estimates of prevalence and incidence of CVD in Greenland do not exist and can only be estimated if cardiovascular diagnoses in the Greenlandic Hospital Discharge Register (GHDR) accurately represent the diagnoses given in the medical records. Since 1987, all inpatient discharge diagnoses from hospitals and local health care centres in Greenland have been registered in GHDR via the personal identification number [11].

The validity of GHDR including completeness and correctness as a whole was addressed in 2011 in an unpublished report to the Greenland Self Rule [12]. The study found a high degree of completeness and correctness of the register. No difference in completeness of the aforementioned variables was found between health care centres, and although completeness varied a little over the studied time period, no systematic differences were detected. There was no difference in correctness of unspecified diagnoses vs. selected specified diagnosis groups, but CVD diagnoses were not validated separately for correctness and could therefore be faulty.

The objective of this study was therefore to validate diagnoses of CVD in GHDR by assessing correctness, defined as the degree to which information in GHDR matched information in medical records.

## Materials and methods

The setting of the study is Greenland, the world’s largest island with a health care system challenged by long distances and remote locations accessible by air or water only when weather conditions allow it. Prescription medicine and all services in the health care system are free of charge. The primary health care system assures the first meeting with the patient, usually with a medical doctor, a nurse or another trained health personnel. It is in the primary health care that preventive and health promoting initiatives take place.

Primary health care is provided in all towns and settlements in Greenland. The health care system in Greenland is divided into 5 health care regions (see Table 1). Each region consists of a number of towns and settlements. A regional health care centre is located in the largest town of the region while local regional health care centres exist in every town. Depending on the number of inhabitants and degree of geographical isolation, the regional and local health care clinics are staffed with physicians, nurses, midwives and other health care workers. In addition to primary health care, some secondary health care and inpatient care take place at the regional and local health care centres. Smaller health stations in settlements are staffed with a nurse or a health care worker depending on the number of inhabitants in the settlements [13]. The secondary health care system undertakes more specialised health care procedures and mostly take place at Queen Ingrid’s Hospital in Nuuk, the only hospital for all of Greenland [13].

**Table 1.** Overview of the health care system in Greenland.

Region	Regional health care centre	Local health care centres	Health care stations in settlements (n)	Total number of health care centres and stations
Avannaa	Ilulissat	Qaanaaq, Upernavik, and Uummannaq	24	28
Disko	Aasiaat	Qasigiannguit and Qeqertarsuaq	9	12
Qeqqa	Sisimiut	Maniitsoq	6	8
Sermersooq	Nuuk^a^	Paamiut, Tasiilaq and Ittoqqortoormiit	8	12
Kujataa	Qaqortoq	Narsaq and Nanortalik	11	14
Total	5	11	58	74
All regions	Queen Ingrid’s Hospital is located in Nuuk and delivers specialised secondary health care to all citizens in Greenland.			

^a^The regional health care centre in Nuuk exclusively delivers outpatient primary health care whereas Queen Ingrid’s Hospital functions as a regional hospital for Nuuk citizens.

### Medical records

Medical records with cardiovascular diagnoses available online were reviewed in this paper for the purpose of assessing the correctness of the information registered in GHDR.

All health care centres and stations (except Queen Ingrid’s Hospital) went online in 2007 with the nationwide implementation of the electronic medical record system Æskulap [14]. Some health care centres had gone online before that, providing information from the years prior to 2007 as well. Older medical records have since then been imported into Æskulap providing records dating back to the beginning of the 1990s in some health care centres. Otherwise, most local health care centres have complete online medical records from 2007 and onwards. Medical records from Queen Ingrid’s Hospital exist in word format on the local hospital drive and can be accessed online by password protected remote desktop access. The process of going from paper to computer was implemented throughout most of Queen Ingrid’s Hospital in 2001. Online accessible medical records from Queen Ingrid’s Hospital are therefore attainable from 2001 and onwards, with a possible exception of some departments that began a little before that. Medical records in paper format were not reviewed in this study.

### The Greenlandic Hospital Discharge Register

GHDR contains discharge diagnoses from admitted patients from Queen Ingrid’s Hospital and all local health care centres in Greenland since 1987. It contains medical information and information on surgical procedures, and it is this register that is validated against medical records for the purpose of assessing the correctness of the information that is documented.

### The Greenlandic Civil Registration System

A personal identification number was assigned to all inhabitants of Greenland alive on 1 May 1972 or later, as a part of the Danish Civil Registration System [11]. We used the personal identification number in the Greenlandic Civil Registration System to link registers and health surveys. Information on sex, place of birth and place of residence are registered. The personal identification number is used for identification and registration in all administrative, social and health contacts.

### Study population

The study population included adult Greenland Inuit invited to participate in one or both population-based health surveys called B99 [15] and Inuit Health in Transition (IHIT) [16] described in detail elsewhere. B99 was conducted in 1999–2001 and IHIT was completed in the years 2005–2010. Register follow-up was approved by the Ethics Committee for Greenland and the Danish Data Protection Agency. As such, every Greenlandic citizen can be followed up in patient registers or cause of death registers in Denmark or Greenland.

### Included diagnoses

We included first time cardiovascular diagnoses according to WHO’s International Classification of Disease 10 [17] if they were either primary or secondary diagnoses. A primary diagnosis describes the diagnosis that was the most resource intensive or serious during the hospitalisation or inpatient visit. Secondary diagnoses refer to conditions that coexist or develop during admission. We included chronic rheumatic heart diseases (I05-I09), hypertensive diseases (I10-I15), ischemic heart diseases (I20-I25), pulmonary heart disease and diseases of pulmonary circulation (I26-I28), other forms of heart disease (I30-I52), cerebrovascular diseases (I60-I69) and diseases of arteries, arterioles and capillaries (I70-I79). We did not include acute rheumatic fever (I00-I02), diseases of veins, lymphatic vessels and lymph nodes, not elsewhere classified (I80-I89) and other and unspecified disorders of the circulatory system (I95-I99). The diagnoses that we did not include were omitted because CVD diagnoses with other underlying risk factors than those monitored by the population surveys were not part of the ethical approval.

### Assessing validity by degree of correctness

Correctness of cardiovascular diagnoses in GHDR in comparison to information in medical records was assessed as agreement between 2 key variables: a diagnosis code and a written diagnosis. If both were present in the medical record, a valid result would be confirmed if there was agreement on both variables. If only 1 variable was present in the medical record, it was considered sufficient for a valid outcome if there was agreement on that variable. If neither a diagnosis code nor a written diagnosis could be found in the medical records, agreement would be assessed based on clinical information, if the clinical information was sufficiently detailed to leave no doubt to the reviewing doctor. In summary, a diagnosis in GHDR was considered valid if a correct diagnosis code, written diagnosis or sufficient relevant clinical information could be found in the medical record. Conversely, a diagnosis in GHDR was considered invalid if an incorrect diagnosis code, written diagnosis or clinical information was found in the medical record.

The data agency approved the study and permission to access GHDR was given by the Agency for Health and Prevention in Greenland. Data was stored on a secure password protected drive at the University of Southern Copenhagen and analysed using SAS 9.4.

## Results

In total, 432 medical records containing a cardiovascular diagnosis according to GHDR were reviewed. Of these, 291 originated from Queen Ingrid’s Hospital representing the years 2001–2013 and 141 came from 11 different local health care centres representing the years 2007–2013. These results translate roughly to 22 CVD diagnoses per year from Queen Ingrid’s Hospital and 20 CVDdiagnoses per year from the 11 local health care centres. We assessed a longer time period for Queen Ingrid’s Hospital because medical records were available online dating further back than medical records from the regional and local health care centres (as described previously).

We found 265 complete medical records from Queen Ingrid’s Hospital, thus 26 missing or incomplete. Similarly, we found 120 complete medical records (21 incomplete or missing) from the local health care centres. Mean age at the time of first CVD diagnosis was similar at 59 years at Queen Ingrid’s Hospital and 63 years at the local health care centres. Sex distribution was also similar with 49.0% male at the local health care centres and 54.5% at Queen Ingrid’s Hospital, see Table 2.

**Table 2.** Results and characteristics of individuals at time of first cardiovascular diagnosis at Queen Ingrid’s Hospital and at 11 health care centres.

Characteristics	Queen Ingrid’s Hospital	Regional and local health care centres (n=11)
Years included	2001–2013	2007–2013
N total	291	141
Complete records, n (missing or incomplete records, n)	265 (26)	120 (21)
Valid outcomes, n (% of complete records)	262 (99)	110 (92)
Invalid outcomes, n (% of complete records)	3 (1)	10 (8)
Males, n (%)	143 (54.5)	59 (49)
Mean age (min-max)	59 (23–96)	63 (35–88)

We found 99% of the CVD diagnoses valid out of 265 reviewed complete medical records from Queen Ingrid’s Hospital in Nuuk. Only 3 invalid diagnoses were found and of these, 2 had disagreement between both diagnosis code and diagnosis and 1 had disagreement on diagnosis alone, because there was no diagnosis code in the medical record. A total of 26 medical records could not be validated for the following reasons: 1 did not contain enough information for validation, 6 could not be looked up using the personal identification number and 19 of the medical records could not be located on the secure drive at Queen Ingrid’s Hospital.

We found 120 complete medical records from 11 local health care centres with 92% of the CVD diagnoses valid. Of the 10 invalid outcomes, 1 was based on disagreement between both diagnosis code and diagnosis, and 7 were based on disagreement between diagnoses alone, because the diagnosis codes were not found in the medical records. The last 2 invalid outcomes were assessed solely on clinical information, because neither a diagnosis code nor a written diagnosis was found in the medical record. A total of 21 medical records could not be validated because of missing or insufficient entries in the medical records.

We reviewed medical records without a cardiovascular diagnosis according to GHDR to assess if a cardiovascular diagnosis should have been given. These 300 records were randomly chosen among those without a CVD diagnosis from the years 2008 to 2013, 150 from Queen Ingrid’s Hospital, 75 from a regional health care centre and 75 from a local health care centre as a representative sample. We found 297 records (99%) correctly registered as non-CVD diagnoses and 3 medical records (1%) with unreported CVD diagnoses.

A second medical doctor double validated 48 of the medical records to assess inter-observer disagreement. These records were chosen semi-randomly by the primary investigator, to include all 11 health care centres and Queen Ingrid’s Hospital and to include roughly 50% of the invalid outcomes (6/13). The second medical doctor was at the time blinded of the results of the primary investigator. The double validation was in complete agreement with the first validation.

We grouped the cardiovascular diagnoses into relevant cardiovascular disease groups to see if invalid outcomes were distributed equally. The invalid outcomes were fairly equally distributed among the CVD groups, see Table 3.

**Table 3.** Frequency of valid and invalid outcomes in the different cardiovascular groups for Queen Ingrid’s Hospital and local health care centres respectively.

		Queen Ingrid’s Hospital		Local clinics	
ICD10 code	CVD group	Valid	Invalid (ICD10)	Valid	Invalid (ICD10)
I01-I09 and I34-I39	Heart valve and rheumatic heart disease	22	0	1	1 (I350)
I10-I15	Hypertension and hypertensive heart disease	44	0	30	4 (I109)
I20-I25	Ischemic heart disease	31	0	19	2 (I219)
I40-I49	Arrhythmias	66	2 (I455, I489)	11	1 (I409)
I50	Heart failure	10	0	10	1 (I500)
I60-I69	Cerebrovascular disease	70	1 (I649)	36	1 (I649)
I26 – I28, I30 – I33, I51-I52, I70-I79	Others	19	0	3	0
Total		262	3	110	10

## Discussion

Overall agreement between cardiovascular diagnoses in the medical records and GHDR was good with 99% and 92% valid CVD diagnoses from Queen Ingrid’s Hospital and local health care centres, respectively, making GHDR suitable for epidemiological use. We found a total of 13 invalid outcomes out of 432 medical records and 3 unreported CVD diagnoses out of 300 medical records, which constitutes an acceptable number of faults.

Although simple in design, a noteworthy strength of this study is the fact that 2 medical doctors got the same results in the double validation of 48 medical records. This gives credibility to the study and provides evidence of internal validity of the results. These results indicate that GHDR is of good quality and may be used for epidemiological purposes.

We did not assess medical records in paper format, but there is no reason to believe that the results may not be inferred to paper records. For both paper and online medical records, diagnostic coding will differ from person to person and place to place and misclassification will be equally likely in both groups. In Greenland, health professionals who see the patients and do the diagnostic registering often come from Denmark, stay for shorter periods of time and often work alone with supervision by phone. These factors may cause changes in diagnostic registering over time, but will not necessarily cause more faulty registrations. We only reviewed medical records for the years 2001–2013 and 2007–2013 for Queen Ingrid’s Hospital and local health care centres respectively, but there is no reason to believe that validity will be markedly different in the years before. There have been no changes in the health care system in Greenland that systematically would affect the diagnostic registering quality of health professionals in the assessed time periods. One event that could affect diagnostic trends is changes in diagnostic classification system, the last one in 1994 when ICD10 was implemented. These changes have been shown to change long-term trends in cause-specific mortality [18].

We found that the invalid outcomes were fairly evenly distributed among CVD groups (Table 3), suggesting non-differential misclassification bias. This supports random errors being made instead of systematic diagnostic mistakes. We did, however, find that Queen Ingrid’s Hospital had fewer (n=3) invalid outcomes than the local health care centres (n=10), although roughly the same number of CVD diagnoses per year, 22 and 20, respectively. The lower number of invalid outcomes may in part be explained by the fact that Queen Ingrid’s Hospital is more specialised and especially internal medicine wards may have more focus on cardiovascular diagnoses and may therefore be more used to diagnosing them. More focus or not, diagnosis of CVD will probably be done more often from now on with the increasing burden of risk factors of CVD, underlining the importance of this study which provides valid data for the estimation of CVD incidence [19].
